# Altered functional connectivity of the thalamus in patients with insomnia disorder after transcutaneous auricular vagus nerve stimulation therapy

**DOI:** 10.3389/fneur.2023.1164869

**Published:** 2023-07-06

**Authors:** Bin Zhao, Yanzhi Bi, Yong Chen, Jinling Zhang, Shuai Zhang, Dongshu Zhang, Peijing Rong

**Affiliations:** ^1^Department of Acupuncture, College of Traditional Chinese Medicine, Southern Medical University, Guangzhou, Guangdong, China; ^2^Department of Psychology, University of Chinese Academy of Sciences, Beijing, China; ^3^Department of Rheumatology and Immunology, Shenzhen People’s Hospital, The Second Clinical Medical College of Jinan University, Shenzhen, Guangdong, China; ^4^Institute of Acupuncture and Moxibustion, China Academy of Chinese Medical Sciences, Beijing, China

**Keywords:** transcutaneous auricular vagus nerve stimulation, functional connectivity, thalamus, insomnia disorder, instant modulation

## Abstract

The pathogenesis of insomnia is related to the dysfunction of the thalamus. Transcutaneous auricular vagus nerve stimulation (taVNS) has proved to be effective in treating insomnia. However, whether taVNS alleviates insomnia through modulating thalamus-related functional connectivity remains unclear. To elucidate the instant modulating effects of taVNS on the resting state functional connectivity (RSFC) of the thalamus, 20 patients with insomnia disorder were recruited to receive taVNS treatment and their resting state functional magnetic resonance imaging (fMRI) data were collected immediately before and after stimulation. The fMRI data were compared with 20 age- and gender-matched healthy subjects who received no stimulation and had RSFC fMRI data collected once. RSFC analyses of the thalamus were performed in both groups. In addition to assessing the group differences between ID patients and healthy controls regarding the RSFC of the thalamus, we examined the taVNS-induced changes of RSFC of the thalamus in ID patients. Before taVNS treatment, the ID patients showed increased RSFC of the thalamus with the right insula and inferior frontal gyrus than healthy controls. After taVNS treatment, the RSFC between the thalamus and the right angular gyrus, left anterior cingulate gyrus, and precuneus were significantly decreased in patients. This study provides insights into the instant brain effects involving the thalamus-related functional connectivity of taVNS performed on insomnia disorder patients.

## Introduction

Insomnia disorder (ID) is characterized by poor sleep quality or insufficient sleep duration, often manifested as difficulty in falling asleep, sleep disorders, waking up early, and other symptoms (1). Approximately 30% of adults experience insomnia symptoms and 10%–20% of the population suffer from chronic insomnia (2, 3). Insomnia is a risk factor for high blood pressure, stroke, anxiety, depression, and weakened immunity (4–7), seriously affecting the quality of life and placing a burden on families and society.

Currently, the treatment for insomnia includes drugs, psychotherapy, behavioral therapy, and physical therapy (8). However, the most sedative sleeping drugs usually cause the body to develop tolerance and dependence, giving rise to impairment of daytime functioning in the short term, and even forming addiction in the long term (9). Transcutaneous auricular vagus nerve stimulation (taVNS) is a neuroregulatory technique which is safer than vagus nerve stimulation (VNS). In the past few years, this technology has been used to treat a variety of diseases such as major depressive disorder (10), epilepsy (11, 12), and mild cognitive impairment (13). It has also proved to be effective in treating insomnia (14, 15). However, the underlying neural mechanisms are not fully clarified.

The thalamus is an important part of the cerebral cortex related to the pathophysiology of insomnia, sleep-wake rhythms, hyperarousal, and emotions (16, 17). Functional impairment of the thalamus and the neural circuits involved in the thalamus are closely associated with insomnia (18–20). Atrophic changes in the thalamus have been demonstrated in ID patients (18). Compared with healthy people, functional connections between the thalamus and some brain regions are abnormal in ID patients, and changes in functional connections with the caudate nucleus, putamen, and hippocampus are negatively correlated with Pittsburgh Sleep Quality Index (PSQI) in ID patients (19). In addition, the thalamus is closely connected to the neocortex by radiating thalamic neuronal fibers (21). In ID patients, the white matter integrity between the thalamus and the frontal lobe is reduced (20), and the thalamo-prefrontal functional junction plays an important role in neurobiological models of insomnia (22). Recently, resting state functional connectivity (RSFC) has been made it possible using whole-brain analysis to identify temporal correlations between brain regions. Consequently, we used seed-based RSFC analysis to investigate different thalamocortical connections between ID patients and healthy matched controls with normal sleep behavior, and we also investigated the different thalamocortical connections pre and post treatment with taVNS. We hypothesized that taVNS modulates specific thalamocortical connections, which may be an important mechanism for the treatment of primary insomnia disorder with taVNS.

## Methods

### Participants

Twenty right-handed adults with insomnia disorder and 20 age-, gender-, and education-matched controls with healthy sleep patterns were included in the present study. All insomnia patients met the diagnostic criteria for insomnia disorder from the Fifth Edition of the American Diagnostic and Statistical Manual of Mental Disorders (DSM-V, 2015), while meeting the following inclusion criteria: (1) Insomnia symptoms duration >3 months; (2) 18–60 years of age; (3) a Pittsburgh Sleep Quality Index (PSQI) score ≥ 5 points; (4) a Hamilton Rating Scale for Depression score < 17 points and a Hamilton Rating Scale for Anxiety score < 14 points; (5) patient stopped taking anti-insomnia medication or other psychiatric medications and receiving acupuncture treatment for nearly 1 month; (6) patient can accept the treatment of taVNS and can undergo magnetic resonance imaging.

### Treatment programs

**Figure 1:**
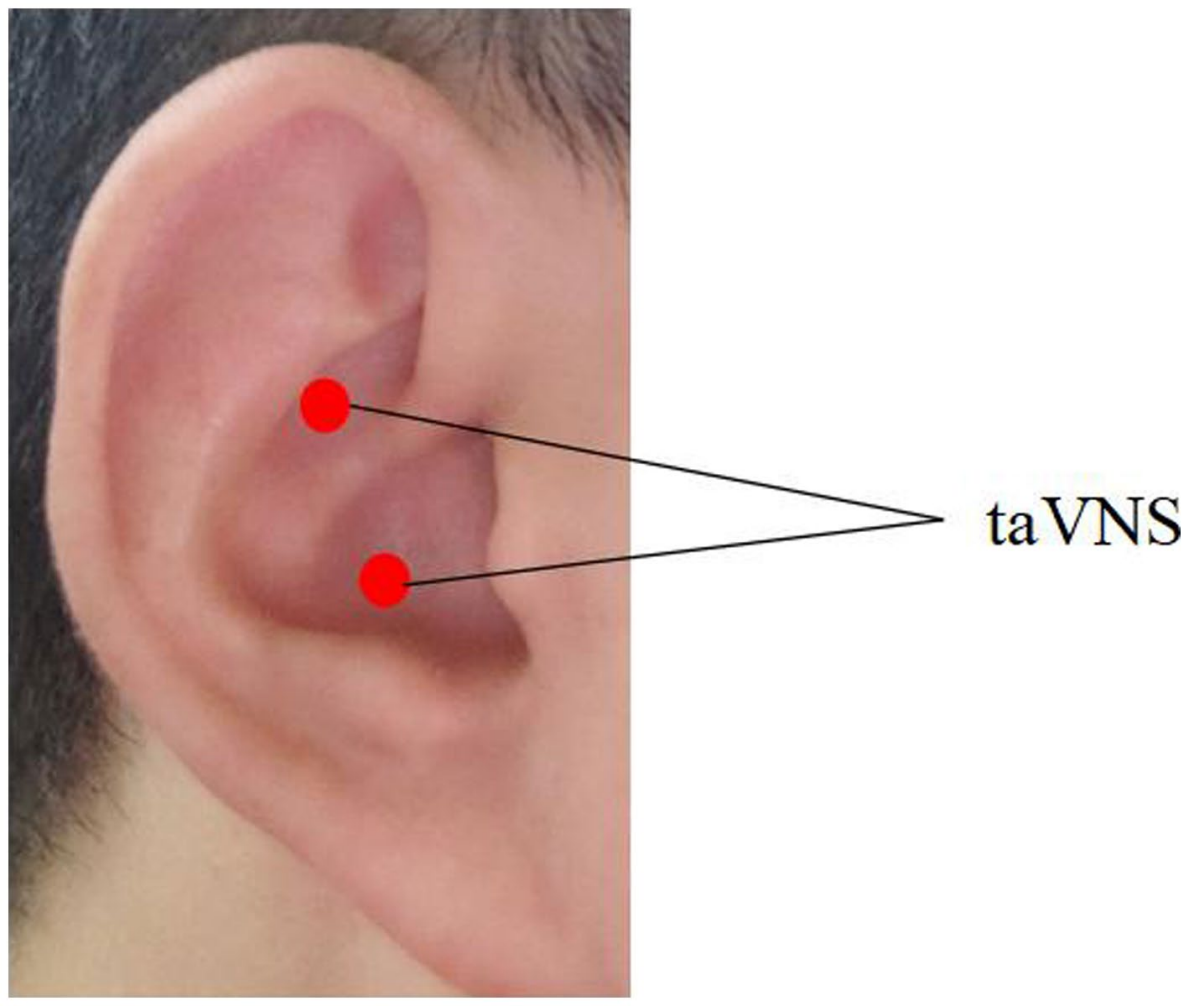
Placement of taVNS stimulation.

The Hwato brand electronic acupuncture instrument (Hwato brand SDZ-IIB) was used for taVNS treatment in ID patients, and bilateral ears were stimulated at the same time (Figure 1). The density wave of pulse frequency was adjusted to 20 Hz (pulse width: 0.2 ms ± 30%). The intensity of stimulation was tolerable to the patients (electrical current ≤50 mA). Each stimulation was conducted for 30 min and the healthy controls were not stimulated.

### MRI data acquisition

MRI data were collected using a 3.0 T MRI system with a standard 20-channel head coil (Siemens Skyra). All subjects underwent conventional structural MRI scans to exclude intracranial organic lesions. Then, a gradient echo-planar imaging sequence was used to acquire the resting-state functional data with the following parameters: TR/TE = 2,500/25 ms, flip angle = 90°, FOV = 240 mm × 240 mm, matrix = 64 × 64, slice thickness/spacing = 3.0/1.0 mm, number of slices = 43. Please note that the MRI data were acquired immediately before and after taVNS treatment in patients. Healthy controls were only scanned once at the time of inclusion.

### Resting-state MRI data analysis

Functional MRI data were analyzed using the Data Processing Assistant for Resting-State fMRI software (DPARSF; http://rfmri.org/DPARSF). The preprocessing steps included: removal of the first 10 volumes, slice timing correction, realignment, spatial normalization to the Montreal Neurological Institute (MNI) space (resampling voxel size = 3 × 3 × 3 mm^3^), spatial smoothing using a Gaussian kernel of 6 mm full width at half maximum (FWHM), detrending to remove the linear drifts, and temporal filtering (0.01–0.08 Hz). Please note that the subjects with head motion of more than 1.5 mm of maximal translation (in any direction of x, y, or z) or 1.5° of maximal rotation throughout the course of scanning were excluded from further analysis.

After preprocessing, the thalamus was selected as the region of interest (ROI) for the functional connectivity (FC) analysis of the resting-state fMRI. The time series of spontaneous brain activity, which were averaged across all voxels of the thalamus, were extracted and correlated with the time series of voxels in the whole brain to produce the individual-level correlation maps. The resulting correlation maps were converted to Z value maps using Fisher’s r-to-z transformation. To investigate the RSFC differences between ID patients and healthy controls, two-sample t tests were conducted. To explore the taVNS treatment induced RSFC changes in ID patients, a paired-sample t test was performed. Statistical maps were thresholded using the GRF theory correction procedure, in which the significance voxel level threshold was set at *p* < 0.001, corrected for multiple comparisons (cluster significance: *p* < 0.05, two-tailed), and a cluster size of over 100 was considered as having significant FC changes.

## Results

### Clinical results: demographic, PSQI, HAMD, and HAMA

The general characteristics of subjects, such as gender, age, and behavioral data, are shown in Table 1. We can see that there is no difference in gender and age (*p* > 0.05), but the scores of the PSQI, HAMD, and HAMA are higher in ID patients than those of healthy controls (*n* = 20:20, *p* < 0.01 or *p* < 0.05).

**Table 1:** Group demographics and clinical measures.

Measure (mean **±** SD)	Participants with insomnia (*n* = 20)	Healthy participants (*n* = 20)	*p*-value
Age, years	35.42 ± 10.72	30.00 ± 6.42	0.067#
Sex (male/female)	7/13	8/12	0.20Δ
PSQI	12.11 ± 3.33	2.45 ± 1.28	*<*0.01#
HAMD	9.42 ± 3.81	2.45 ± 2.35	*<*0.01#
HAMA	6.84 ± 4.49	2.50 ± 3.07	*<*0.05#

SD, standard deviation; PSQI, Pittsburgh Sleep Quality Index; HAMD, Hamilton Rating Scale for Depression; HAMA, Hamilton Rating Scale for Anxiety. # Indicates *p*-values for two-sample *t*-tests. ΔIndicates *p*-values for Chi-square test.

### The thalamocortical RSFC differences between insomnia disorder patients and healthy controls

**Figure 2:**
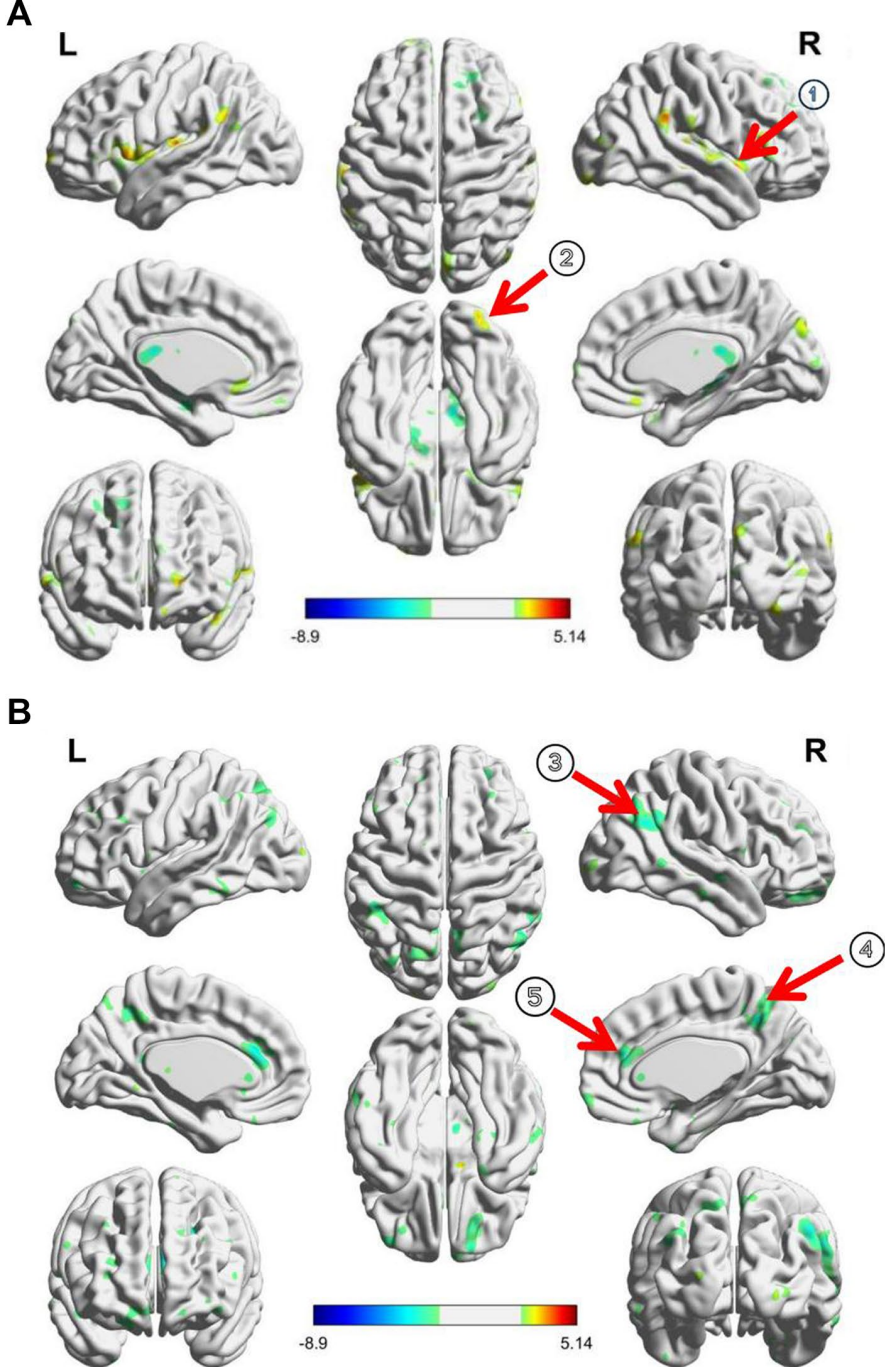
**(A)** RSFC changes between PI patients and healthy controls before taVNS treatment; **(B)** taVNS treatment induced RSFC changes of thalamus in patients; ① Insula, ② inferior frontal cortex, ③ precuneus, ④ angular cortex, and ⑤ anterior cingulate cortex.

Compared to the healthy controls, the ID patients showed an increased RSFC of the thalamus with the right insula and inferior frontal gyrus (GRF correction, Table 2; Figure 2A).

**Table 2:** RSFC differences between insomnia disorder patients and healthy controls before taVNS treatment, as well as the taVNS treatment-induced RSFC changes of the thalamus.

Brain regions	Side	Peak			Cluster size	*t-*value
**Patients-pre > HC**						
Insula	Right	39	3	−6	161	4.8275
Inferior frontal cortex					140	
**Patients-pre > patients-post**						
Angular cortex	Right	54	–57	30	214	−3.9474
Anterior cingulate cortex	Left	−15	33	6	125	−5.4416
Precuneus	Right	0	−54	48	150	−4.687
Precuneus					182	

### The taVNS treatment induced thalamocortical RSFC changes in insomnia disorder patients

After taVNS treatment, the RSFC between the thalamus and the right angular gyrus, anterior cingulate gyrus, and precuneus were significantly decreased in ID patients (GRF correction, Table 2; Figure 2B).

## Discussion

Compared with healthy people, the level of glucose metabolism in the whole brain increases in ID patients during sleep and wakefulness, indicating that patients with insomnia disorder have high arousal in the whole brain (23)_._ The thalamus is a relay station for various senses. The fibers from the brain stem reticular ascending activation system can transmit various stimulatory signals *in vivo* and *in vitro* to the dorsal thalamic nucleus, and then project them to a wide area of the cerebral cortex to maintain the awakening state of the cerebral cortex. Therefore, we selected the bilateral thalamus as the seed point to analyze the changes in functional connections between it and all brain regions. In the study, we found that, in ID patients, there were abnormal functional connections between the thalamus and the salient network, negative emotion network, and default mode network (DMN), and taVNS could effectively modulate these abnormal functional networks and brain regions.

### RSFC changes between the thalamus and the salient network and negative emotion network

The anterior cingulate gyrus and bilateral insula are important nodes of the salient network (24, 25), whose main functions are to monitor, identify, and conduct preliminary analysis of external stimuli. Previous studies have shown that, in ID patients, the activity of some brain regions within the salient network is weakened (26, 27), while FC is increased between brain regions within the salient network, such as between the dorsal and ventral anterior insula (28), and between the anterior insula and bilateral anterior thalamus (29). Our study also confirmed that FC was increased between the bilateral thalamus and insula in ID patients. We hypothesize that this increased FC within the salient network is used to compensate for impaired monitoring and recognition in ID patients due to the decreased salient network activity. Although we did not observe a regulatory effect of taVNS on the functional connections between the thalamus and insula, our study also found that after taVNS treatment in ID patients, FC decreased between the bilateral thalamus and the anterior cingulate gyrus. The insula and dorsal anterior cingulate gyrus are the main components of the negative emotional network, which is closely related to the amygdala. It has been shown that functional connections between the amygdala and insula and also the left amygdala and the anterior cingulate gyrus are weakened in insomnia disorder patients (30, 31). The abnormal negative emotional network will affect the brain’s processing and management of negative emotions, which can explain the common anxiety and depression symptoms in ID patients. Our previous studies have also confirmed the regulation effect of taVNS on negative emotions in ID patients with depression (32), and the activation state of the insula lobe can be used as a predictor of the efficacy of taVNS (33). Therefore, taVNS can improve the negative emotional symptoms often associated with insomnia disorder patients using the salient network and the negative emotional network’s processing and management of negative stimuli. It may be an important mechanism to relieve insomnia symptoms.

### RSFC changes between the thalamus and DMN

The results of this study showed that, compared with normal subjects, FC increased between the thalamus and the right inferior frontal gyrus in ID patients with insomnia disorder. The right inferior frontal gyrus is related to executive control, attention, and self-awareness. In ID patients, the FC in the frontoparietal network is weakened (34). We speculate that this is a compensatory mechanism for the low function of the frontoparietal network in ID patients. At resting state, the frontoparietal network participates in DMN function regulation (35). The presence of self-referential load in cognitive content, such as rumination and anxiety, is likely to be associated with a disruption or imbalance in the DMN (36). During passive tasks (cognitive-behavioral therapy for insomnia), ID patients were able to decrease DMN activity (36). Meanwhile, the corticostriate-thalamic-cortex circuit is interconnected with the frontal cortex, so cortical hyperarousal can be modulated by filtering thalamic sensory input (37). In our study, after short treatment with taVNS, the FC between the thalamus and the right angular cortex, left anterior cingulate cortex, and precuneus, which are hubs of the DMN in ID patients, decreased compared with that before stimulation, suggesting that taVNS can reduce the excessive arousal state of the brain region of the DMN caused by the thalamus in ID patients, which may be an important mechanism for treating primary insomnia.

### Limitations

There were several limitations associated with the study. First, the sample size of the ID group was small. Since the study only observed the immediate effect of taVNS on ID, and did not involve the evaluation of efficacy, we referred to the previous sample size requirements of functional MRI, but we will further expand the sample size in future studies. Second, we did not set a spurious stimulus control group; although it was the only intervention, other effects on the FC changes between pre- and post-taVNS cannot be excluded. Third, we only observed the immediate effects of taVNS, and there was no comparison of brain activity after long-term treatment. Nevertheless, the present study provides the resting-state data of ID patients who underwent taVNS, and these data can be used as grounds for future research.

## Conclusion

The functional connectivity changes between the thalamus and the right angular gyrus, left anterior cingulate gyrus, and precuneus are the basis for the treatment of insomnia disorder and emotional and cognitive disorders caused by insomnia disorder through taVNS.

## Data availability statement

The raw data supporting the conclusions of this article will be made available by the authors, without undue reservation.

## Ethics statement

The studies involving human participants were reviewed and approved by the Ethics Committee of the Acupuncture and Moxibustion Institute of the Chinese Academy of Chinese Medical Sciences. The patients/participants provided their written informed consent to participate in this study.

## Author contributions

PR and DZ contributed to the design of the study. BZ, JZ, and SZ contributed to the data acquisition. BZ and YB contributed to the data analysis. BZ, SZ, DZ, and PR contributed to the results interpretation. BZ, YB, YC, SZ, and PR contributed to manuscript preparation. All authors contributed to the article and approved the submitted version.

## Funding

This work was supported by the National Natural Science Foundation of China (82004174) and the Project of Traditional Chinese Medicine of Southern Medical University Youth Program.

## Conflict of interest

The authors declare that the research was conducted in the absence of any commercial or financial relationships that could be construed as a potential conflict of interest.

## Publisher’s note

All claims expressed in this article are solely those of the authors and do not necessarily represent those of their affiliated organizations, or those of the publisher, the editors and the reviewers. Any product that may be evaluated in this article, or claim that may be made by its manufacturer, is not guaranteed or endorsed by the publisher.
